# Bidirectional regulation of osteogenic differentiation by the FOXO subfamily of Forkhead transcription factors in mammalian MSCs

**DOI:** 10.1111/cpr.12540

**Published:** 2018-11-05

**Authors:** Duanjing Chen, Yuanyuan Gong, Ling Xu, Mengjiao Zhou, Jie Li, Jinlin Song

**Affiliations:** ^1^ College of Stomatology Chongqing Medical University Chongqing China; ^2^ Chongqing Key Laboratory for Oral Diseases and Biomedical Sciences Chongqing China; ^3^ Chongqing Municipal Key Laboratory of Oral Biomedical Engineering of Higher Education Chongqing China

**Keywords:** FOXO, mesenchymal stem cells, microRNA, osteogenic differentiation, post‐translational modification

## Abstract

Through loss‐ and gain‐of‐function experiments in knockout and transgenic mice, Forkhead box O (FOXO) family transcription factors have been demonstrated to play essential roles in many biological processes, including cellular proliferation, apoptosis and differentiation. Osteogenic differentiation from mesenchymal stem cells (MSCs) into osteoblasts is a well‐organized process that is carefully guided and characterized by various factors, such as runt‐related transcription factor 2 (Runx2), β‐catenin, osteocalcin (OCN), alkaline phosphatase (ALP) and activating transcription factor 4 (ATF4). Accumulating evidence suggests multiple interactions among FOXO members and the differentiation regulatory factors listed above, resulting in an enhancement or inhibition of osteogenesis in different stages of osteogenic differentiation. To systematically and integrally understand the role of FOXOs in osteogenic differentiation and explain the contrary phenomena observed in vitro and in vivo, we herein summarized FOXO‐interacting differentiation regulatory genes/factors and following alterations in differentiation. The underlying mechanism was further discussed on the basis of binding types, sites, phases and the consequent downstream transcriptional alterations of interactions among FOXOs and differentiation regulatory factors. Interestingly, a bidirectional effect of FOXOs on balancing osteogenic differentiation was discovered in MSCs. Moreover, FOXO factors are reported to be activated or suppressed by several context‐dependent signalling inputs during differentiation, and the underlying molecular basis may offer new drug development targets for treatments of bone formation defect diseases.

## 1 INTRODUCTION

As osteoblasts are short‐lived and in need of being constantly replenished with new cells to maintain the synthesis of bone matrix, their mesenchymal stem cells (MSCs) are considered to play essential roles in bone regeneration. By controlling proliferation, self‐renewal and differentiation, MSCs maintain a balance between bone formation and bone resorption.^1^ Several studies have revealed that bone formation defects may result from the decline of MSCs osteogenic differentiation.^2^, ^3^, ^4^

In C *elegans*, abnormal dauer formation protein 16 (DAF‐16) functions in determining the lifespan via mediating metabolic adaptation and resistance to oxidative stress.^5^ In mammals, FOXO is the vertebrate orthologue of DAF‐16.^6^ Adult tissues maintain their regenerative capacity by the proliferation and differentiation of a range of stem cells. Thus, *FOXO* genes presumably function in maintaining the self‐renewal and differentiation capacity of stem cells. Indeed, a plethora of studies have uncovered essential roles of FOXO transcription factors along stages of osteogenic differentiation in MSCs. When FOXO‐regulated osteogenic differentiation in MSCs is disrupted, constitutive oxidative stress and inhibited differentiation may lead to bone formation defect diseases, such as delayed bone fracture healing and osteoporosis, an ageing‐related bone loss disease.^7^, ^8^ Furthermore, MSC‐based therapy has yielded encouraging outcomes in the treatment of bone formation defect diseases.^4^ Therefore, finding the mechanism of FOXO‐mediated MSCs differentiation may provide another promising drug development target to antagonize bone formation defect diseases. In this review, we summarized the roles of FOXO transcription factors played in periods of osteogenic differentiation, from initiation to lineage commitment to terminal differentiation.

## 2 STRUCTURAL AND FUNCTIONAL RELATIONSHIPS OF FOXO ISOFORMS

The FOXOs belong to the forkhead transcription factor family, which is characterized by a winged–helix DNA‐binding motif (the so‐called forkhead domain). In mammalian cells, four FOXO isoforms have been identified and characterized: FOXO1 (FKHR), FOXO3 (FKHRL1), FOXO4 (AFX or Mllt7) and FOXO6.

### 2.1 Function of FOXO family members in mammalian stem cells

FOXO family members are reported to play essential roles in various stem cells and their located systems, such as regulating proliferation and self‐renewal capacity of neural stem cell (NSC),^9^, ^10^ and intracellular oxidative stress and cell number of hematopoietic stem cells (HSCs)^11^ and osteoblasts.^12^, ^13^ Besides, cell homoeostasis, cellular survival, cell‐cycle arrest, autophagy, inflammation and cell differentiation have also been shown to be affected by FOXO transcription factors. Moreover, alterations in FOXO‐regulated transcription are closely related to human diseases, such as bone formation defect diseases, as mentioned above.

### 2.2 Distribution and function of FOXO isoforms

FOXOs are relatively ubiquitously expressed, with FOXO1 expression being the highest in the adipose tissue, liver and bone.^14^, ^15^, ^16^ FOXO3 is predominantly expressed in the heart, brain, kidney, ovary and bone.^10^, ^16^ FOXO4 shows the highest expression in the muscle, heart and bone.^14^, ^15^, ^16^ FOXO6 appears to be uniquely expressed in the brain, liver and oxidative muscle.^17^, ^18^, ^19^ Nevertheless, FOXO1, FOXO3 and FOXO4 are all expressed in bone cells.^12^, ^20^

Through loss‐ and gain‐of‐function experiments in transgenic and knockout mice, mice lacking the *FOXO* alleles were shown to display remarkably different phenotypes.^21^ Deletion of *FOXO1* is lethal due to defective angiogenesis^21^ but protects against insulin resistance, while overexpressing a constitutively active *FOXO1* in the liver leads to diabetes.^22^ *FOXO3^‐/‐^* mice exhibit lymphoproliferation and widespread organ inflammation due to hyperactivated helper T cells.^21^ And their isolated NSCs show decreased numbers and the capability of self‐renewal and differentiation.^10^ *FOXO3* ablation in female mice exhibits premature ovarian failure, resulting from quicken differentiation and early depletion of ovarian follicles.^21^, ^23^ *FOXO4^‐/‐^* mice are shown more susceptible to colitis, indicating that FOXO4 also plays a role in intestinal mucosal immunity.^24^ *FOXO6^‐/‐^* mice display decreased hepatic glucose production, enhanced insulin sensitivity^25^ and decreased dendritic spine density in hippocampal neurons.^18^

## 3 FOXOS IN OSTEOGENIC DIFFERENTIATION

### 3.1 Overview of osteogenesis in MSCs

**Figure 1:**
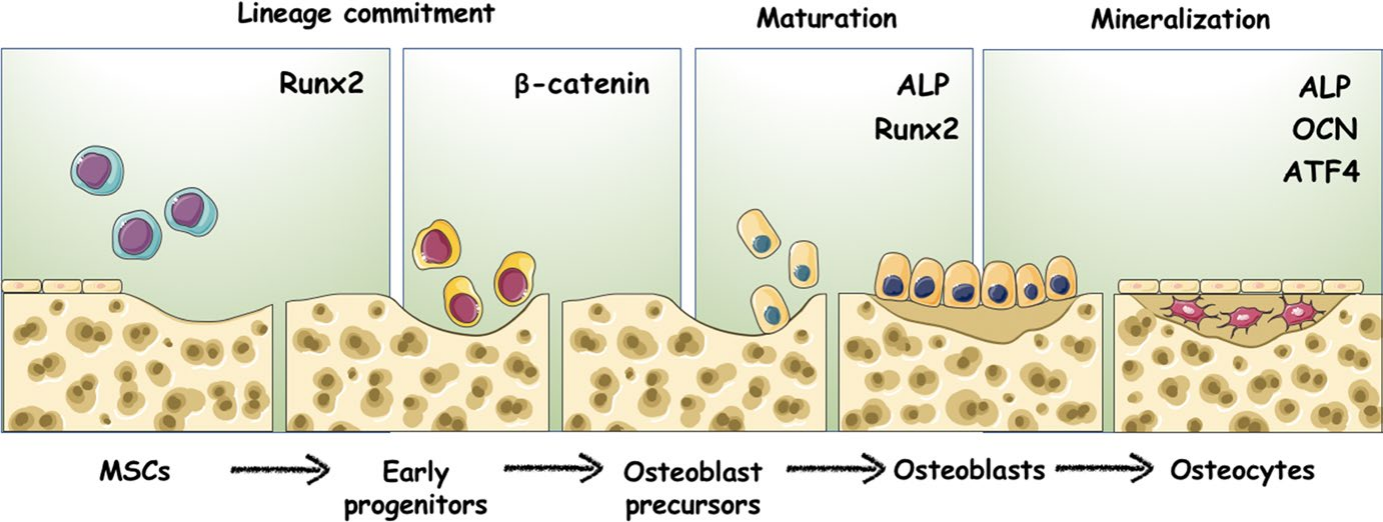
Regulatory factors take part in different stages of osteogenic differentiation. At the early stage, Runx2 and β‐catenin determine the lineage of osteoblasts from multipotent MSCs. Then, the maturation of the osteogenic lineage is characterized by the activity and expression of ALP. Mature osteoblasts will become entombed in osteoid and synthesize OCN and bone matrix, which are regulated by ATF4

MSCs are multipotent stromal cells that are capable of self‐renewal and multilineage differentiation.^26^ MSCs function as precursors to a variety of mature mesenchymal cell types, including adipocytes, myocytes, chondrocytes and osteoblasts. Different stages of differentiation are guided by multiple extracellular signals and transcription factors. In osteogenic differentiation, the expression of runt‐related transcription factor 2 (Runx2) and nuclear translocation of β‐catenin are required at the early stage^27^ (Figure 1). The development of a committed osteoblast precursor into a mature one can be characterized by the activity and expression of alkaline phosphatase (ALP) and categorized into the phase of maturation.^27^ In turn, mature osteoblasts will become entombed in osteoids to become osteocytes. Osteocytes synthesize osteocalcin(OCN) and bone matrix, which are mediated by β‐catenin, to initially form the bone mass and later function in bone remodelling and mineral metabolism.^28^

Runx2, ALP and OCN are integral factors for the control of osteogenesis, and their transcriptional network may be silenced or enhanced by interruptions from other transcription factors, such as FOXOs.

### 3.2 The roles of FOXO isoforms played in the osteogenesis of MSCs in transgenic mice models

By interrupting osteogenic differentiation, mice lacking the *FOXO* alleles in osteoblastic progenitors (including early progenitors and committed osteoblast precursors) and mature osteoblasts were revealed to display a remarkably high bone mass phenotype^29^ or an osteoporosis phenotype^12^, ^20^ in different studies via loss‐ and gain‐of‐function experiments in transgenic mice. In Iyer's experiment, triple *FOXO* deletion in committed osteoblast precursors of Osx1‐Cre mice yielded increased spinal and femoral bone mineral density (BMD), cancellous and cortical bone mass, and cortical thickness.^29^ In contrast, triple *FOXO* deletion in Mx‐Cre^+^ mice early progenitors was followed by an increase in cellular oxidative stress and osteoblast apoptosis as well as a decrease in the number of osteoblasts, the rate of bone formation, and cancellous and cortical bone mass.^12^, ^13^ Taken together, these results imply that FOXO factors may have different functions at distinct stages of osteoblast lineages along osteogenesis.

FOXOs are relatively ubiquitously expressed, with FOXO1, FOXO3 and FOXO4 all expressing in bone cells.^12^, ^29^ Analysis of the expression of the three FOXO isoforms in primary osteoblasts showed that FOXO1/3 were the abundantly expressed members among them.^29^, ^30^ To determine whether deletion of *FOXO1* or *FOXO3* alone in osteo‐progenitors or mature osteoblasts could recapitulate the effect of triple FOXO deletion, *FOXO1* or *FOXO3* ablation mice models were constructed by Rached et al and Lyer et al, respectively. In vivo experiments showed that *FOXO3* deficiency in both committed osteoblast precursors and mature osteoblasts failed to affect any of these osteogenic‐related measurements.^20^, ^29^ Interestingly, overexpression of *FOXO3* in mature osteoblasts decreased oxidative stress and osteoblast apoptosis, and increased osteoblast number, bone formation rate and vertebral bone mass,^12^, ^31^ indicating a FOXO3 threshold‐dependent relationship on mature osteoblasts. Thus, the influence of low‐level FOXO3 on both progenitors and mature osteoblasts is limited, while significant enhancement is observable when expression reaches to a high level at mature osteoblasts.

However, there is almost no studies constructing mice model of *FOXO1* ablation in osteoblastic precursors but in mature osteoblasts. The mice, ablation of *FOXO1* in mature osteoblasts, were characterized by consequent decreased osteoblast numbers, bone mineral density, bone formation rate and bone volume,^20^ implying a more important role of FOXO1 in late stage of osteogenic differentiation than FOXO3. FOXO1 was found to be expressed at the highest expression levels in areas of MSC differentiation into bone, such as the developing calvaria (sevenfold) and diaphysis (sevenfold) of long bones,^32^ implying FOXO1 might participate in modulating osteogenic differentiation. More evidences were found to support this hypothesis; FOXO1 mRNA levels were observed to increase by twofold with BMP‐2 treatment, which was followed by an increase in the reporter activity of an *ALP* promoter construct and prevented stem cells from differentiating into fat or muscle cells.^33^, ^34^, ^35^, ^36^ This is consistent with the results that FOXO proteins could suppress the expression and transcriptional activity of PPAR, a potent repressor of osteogenesis.^37^ In addition, FOXO1 activity was proved upregulated during diabetic fracture healing, particularly in chondrocytes^38^ and MSCs,^39^ indicating an antagonizing role of FOXO1 in insulin‐delayed bone regeneration. Generally, in vivo studies focusing on the individual role of FOXO1 in osteogenic differentiation are still inadequate.

In sum, FOXO1, FOXO3 and FOXO4 are all expressed and function in bone cells. Generally, they may get together to play a bidirectional regulatory role in stages of bone formation. Respectively, it seems that FOXO3 alone may fail to influence the differentiate capacity of committed osteoblast precursors, while either one of FOXO1 and high‐level FOXO3 could enhance osteogenic differentiation at the late stage. However, the in vivo functions of FOXO1 in early progenitors and committed osteoblast precursors, FOXO3 in early progenitors and FOXO4 in osteoblast lineages all have merely been reported thus far.

### 3.3 Mechanisms underlying the regulation of osteogenic differentiation by FOXO members

What is the molecular basis underlying the ability of FOXOs to regulate osteogenic differentiation? Like most transcription factors, FOXO factors mediate disparate transcriptional programs by direct DNA binding or as part of protein complexes capable of regulating context‐dependent programs of gene expression that affect osteogenic differentiation. As transcription factors, FOXO proteins bind to the promoters of *Runx2*,^40^ *ALP* 41and *OCN*.^30^ To form protein complexes, FOXO factors have also been shown to bind to β‐catenin^42^ and ATF4.^20^ Depending on their binding partners, FOXO factors can act as direct or indirect transcriptional activators or repressors. Such protein complexes may be mimicked by chromosomal translocations that result in transcripts that encode a transcriptional activation domain from FOXO fused to a different DNA‐binding domain. For example, one such translocation associated with bone regeneration results in a fusion of the genes encoding the transcription factors ATF4 and FOXO1, which produces transcripts containing the intact ATF4 DNA‐binding domain and the FOXO1 transcriptional activation domain.^20^

In addition, FOXO members are able to bind to microRNAs, which are not specific to any differentiation stage but can nonetheless regulate the differentiation process by acting as downstream regulators of other pathways.

Taken together, these results suggest that by directly binding DNA or forming a complex, FOXO factors interacted with differentiation regulatory factors and thereby play critical roles in the regulation of bone formation.

### 3.4 The FOXO members mediate the stages of osteogenic differentiation

#### 3.4.1 FOXO deficiency initiates osteogenic differentiation in MSCs

MSCs are known to have low levels of intracellular ROS due to the high expression of antioxidant‐like glycolysis to manage oxidative stress.^43^ Compared with MSCs, more committed osteoblast precursors were observed to have higher levels of antioxidant enzymes, such as Mn‐SOD and catalase, indicating an increase in intracellular ROS levels during differentiation.^44^ This phenomenon was paralleled with a metabolic switch from glycolysis to enhanced mitochondrial respiration to ensure a sufficient energy supply to complete differentiation.^45^ Additionally, the perinuclear arrangement of mitochondria determined stem cell differentiation competence.^46^ Thus, increased ROS levels may act as an intracellular signal to drive MSCs to exit from quiescence and result in the activation of a genetic program triggering lineage commitment.^47^, ^48^ This phenomenon is consistent with Almeida's theory that an acute increase in ROS may transiently stimulate osteogenic differentiation.^49^ As FOXOs are required for MSCs to maintain low levels of intracellular ROS, increased oxidative stress would require either a reduction in FOXO levels or a phosphorylation‐induced inhibition of their transcriptional activity. This phenomenon is supported by Wu.’s study reporting that ablation of *PTEN* (antagonizing PI3K function) in bone marrow‐derived stem cells (BMMSCs) results in the phosphorylation and inhibition of FOXO isoforms via enhancing Akt activation.^50^ It is entirely possible that the debilitating effects in the MSCs compartments observed in these mice are due to reduced FOXO activity, resulting in increased oxidative stress that drives differentiation.

#### 3.4.2 FOXO1/3 assist in osteogenic lineage commitment by binding to Runx2

Runx2 has been shown to be crucial for osteogenic differentiation in numerous studies by determining the lineage of osteoblasts from multipotent MSCs, triggering the expression of major bone matrix protein genes in early progenitors^51^, ^52^ and keeping the osteoblasts in an immature stage at the late stage of differentiation.^52^ Throughout differentiation, Runx2 regulates the expression of various osteoblastic genes such as *type Ⅰ collagen* (in early progenitors), *osteopontin* (in immature osteoblasts) and *osteocalcin* (*Bglap2* gene, in mature osteoblasts).^52^ Runx2 mRNA levels were decreased as a result of triple *FOXO* deletion in BMMSCs.^12^ In Siqueira's and Moriishi's studies, retroviral introduction of either FOXO1 or FOXO3 shRNA into MC3T3‐E1 cells (an early osteoblast progenitor cell line) sharply reduced the upregulated expression of Runx2 and OCN during osteogenic differentiation, even in the presence of strong osteogenic stimulants.^53^, ^54^ This result is in line with the significantly increased protein expression of Runx2 in response to *FOXO1* overexpression.^40^ Taken together, the above‐mentioned results of loss‐ and gain‐of‐function experiments indicate that FOXO1 and FOXO3 act as upstream regulators of Runx2 throughout differentiation.

**Figure 2:**
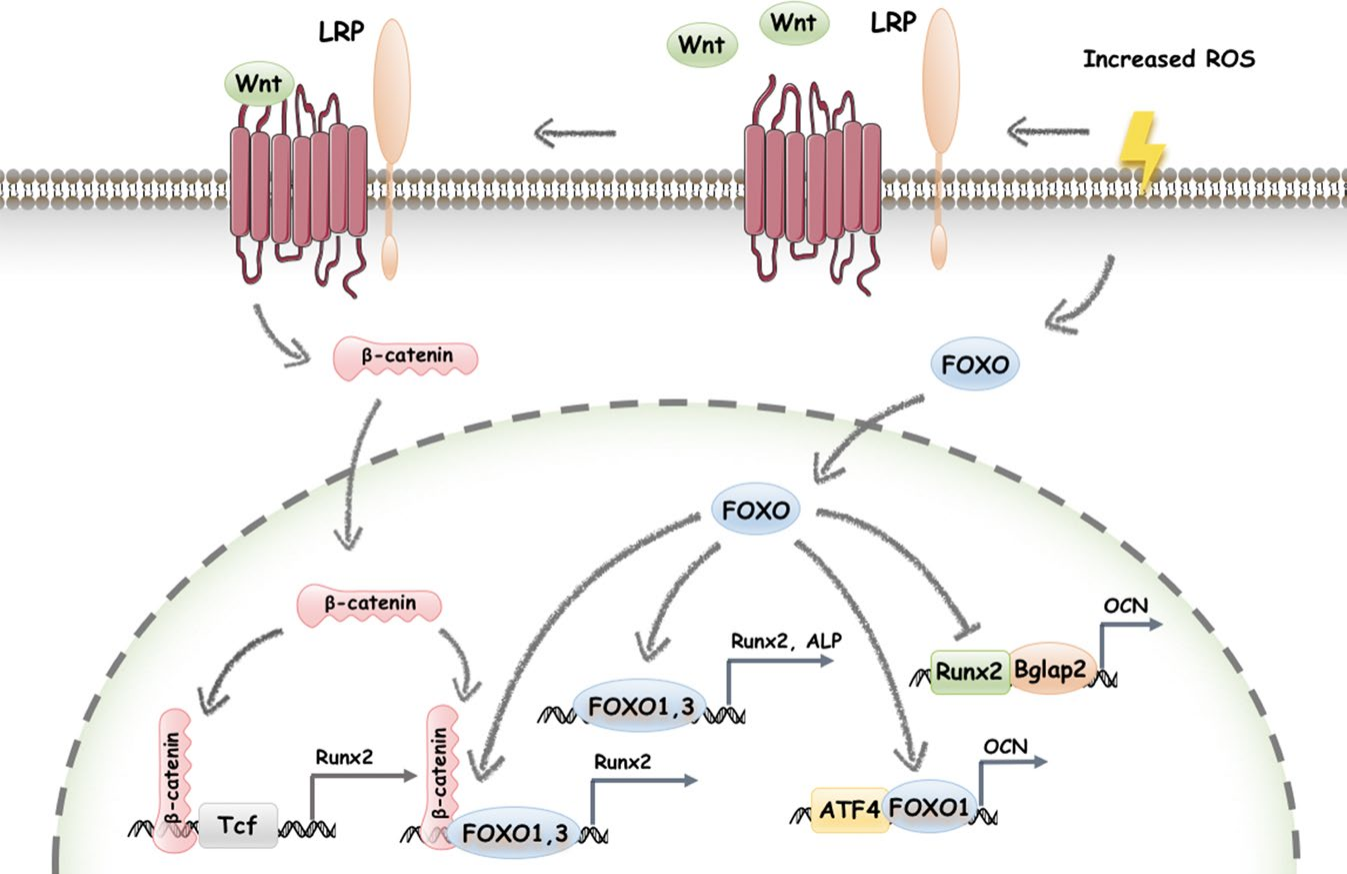
The FOXOs mediate the stages of osteogenic differentiation. The debilitating effects of FOXO may lead to excessive oxidative stress, which then drives MSCs to exit from quiescence and initiates differentiation. FOXO1 directly targets the *Runx2* promoter to induce promoter activity and Runx2 protein expression, determining the lineage commitment and upregulating the expression of more osteoblastic genes. Increased ROS activates Wnt, which binds to the Frizzled‐LRP5/6 receptor complex. As a result, cytosolic β‐catenin translocates into the nucleus to bind with and activate Tcf, inducing osteogenesis‐related target gene transcription. By binding directly to β‐catenin, FOXOs divert β‐catenin from Tcf‐ to FOXO‐mediated transcription and attenuate osteogenesis in this way. Moreover, FOXO1 binds to the *ALP* promoter to promote ALP expression and activity, which are regarded as characteristics of osteoblast precursors maturation. As *Bglap2* is a target gene of Runx2, FOXO1 downregulates Runx2‐dependent transcriptional activity on Bglap2. Stress signals stimulate the translocation of the FOXO1 and ATF4 complex into the nucleus, enhancing ATF4‐dependent OCN expression, GSH and collagen synthesis, and mineralization

FOXO1 was confirmed to directly target the *Runx2* gene during osteoblast differentiation (Figure 2) via chromatin immunoprecipitation (ChIP) assays performed by van der Horst et al.^41^ Via sequence analysis, three putative FOXO1 binding sites on *Runx2* promoter were further discovered; one was between −900 and −1300 kb, and two were below the −900 kb region .^40^ This result also revealed that the association between FOXO1 and Runx2 occurs during the early stages (48 hours) of differentiation in C3H10T1/2 cells,^40^ and middle stage (days 7 and 14) of differentiation in MC3T3‐E1 cells.^53^ In addition, as Runx2 is a predominant factor at the early stage, it seems that FOXO1/3 may play a promoting role in early progenitors.

#### 3.4.3 FOXO1/3/4 inhibit osteogenic lineage commitment by competitive conjunction with Tcf to β‐catenin

Via mediating Wnt signal transduction, β‐catenin is an essential factor for the commitment of early progenitors to osteoblast precursors.^55^, ^56^, ^57^, ^58^ A decade ago, it was shown that ROS‐induced FOXO activation and subsequently FOXO‐mediated transcription also require binding of β‐catenin,^42^ and they physically interact in bone cells. High‐ROS‐stimulated FOXO‐luc activity was attenuated by a reduction of β‐catenin, which confirmed the requirement of β‐catenin for FOXO‐mediated transcription. When ROS levels are maintained in balance and Wnt receptors activation is absent, cytosolic β‐catenin is usually degraded via the proteasome system. Once Wnt binds to the Frizzled‐LRP5/6 receptor complex, cytosolic β‐catenin becomes stabilized and translocates into the nucleus to bind with and activates the transcription factor Tcf and induces the transcription of Wnt/β‐catenin/Tcf target genes, such as *Axin2* as well as increasing the expression of osteogenic‐related genes, including *Runx2*,* distal‐less homeobox 5* (*Dlx5*), *osteoprotegerin* (*OPG*) and *osterix* (*Osx*).^59^, ^60^, ^61^ However, high‐ROS levels or growth factor depletion suppresses Tcf‐mediated transcription in a FOXO‐dependent manner.^49^, ^62^, ^63^ β‐catenin was also found to directly bind to FOXO and partly enhance FOXO transcriptional activity like increasing expression of cell‐cycle inhibitor p27Kip1 and a consequent arrest in G_1_.^42^ However, whether a similar enhancement could be observed in FOXO‐regulated expression of osteogenic factors such as Runx2 remains unclear. As both FOXO‐ and Tcf‐mediated transcription may utilize a limited pool of available β‐catenin, the diversion of β‐catenin from Tcf‐ to FOXO‐mediated transcription may be responsible for the decrease in bone formation (Figure 2).^29^, ^42^ In another word, the antiosteogenic actions of FOXOs result from the binding of FOXOs to β‐catenin at lineage commitment stage. And it is in consistent with the high bone mass phenotype displayed after FOXO triple deletion in osteoblast precursors in vivo, as mentioned in section 3.2.

As important transcription factor or cofactors, the FOXO family and β‐catenin could both be deacetylated by sirtuin1 (Sirt1), which is an NAD^+^‐dependent deacetylase, to unleash Wnt signalling and promote bone formation.^64^, ^65^ By decreasing FOXO3 levels and deacetylating FOXO1, Sirt1 attenuates the association between β‐catenin and FOXO in osteoblast precursors, leading to a decrease in diversion of β‐catenin from Tcf‐ to FOXO‐mediated transcription and an increase in bone formation.^66^, ^67^ Besides Sirt1‐mediated disruption, Akt activator might also override the effect of ROS on the interaction between FOXOs and β‐catenin by phosphorylating FOXOs.^68^

#### 3.4.4 FOXOs promote ALP gene transcription at maturation stage of differentiation

Mineralization is the process by which hydroxyapatite is deposited in the extracellular matrix. Alkaline Phosphatase (ALP), a membrane‐bound metalloenzyme, hydrolyses pyrophosphate and provides inorganic phosphate to promote mineralization physically. The activity and localization of ALP are valuable indexes for tissue development and differentiation, and ALP has become the most clinically relevant enzyme in the diagnosis of bone diseases. Triple FOXO deletion in BMMSCs results in decreased expression of ALP.^12^ In Siqueira's and Moriishi's studies, knockdown of *FOXO1* or *FOXO3* in osteo‐progenitors sharply reduced the upregulated expression of ALP during osteogenic differentiation, indicating that FOXO1 and FOXO3 act as upstream regulators of ALP .^53^, ^54^ Consistently, when *FOXO1* was overexpressed, ALP expression was observed to increase significantly in response.^40^ BMP‐2‐induced FOXO1 transcription also increased the reporter activity of an *ALP* promoter construct.^53^ Additionally, FOXO3a‐TM, which is a constitutively active form of FOXO3, also increases bone mass, ALP activity and mineralization.^12^, ^54^ Via ChIP assays, FOXO1 directly targeting the *ALP* gene was further confirmed.^41^ Taken together, these results concluded that FOXO1 and FOXO3 upregulate the expression and activity of ALP (Figure 2).

#### 3.4.5 FOXOs mediate OCN gene transcription in later stage of differentiation

Osteocalcin (OCN), which is produced by mature osteoblasts, is often used as a marker of the stage division of osteogenic differentiation and has dual roles in osteogenesis. OCN is not only implicated in bone mineralization, calcium ion homoeostasis and body's metabolic regulation but also prevents excessive mineralization via slowing down crystal growth, indicating dual roles of OCN on osteogenesis. Generally, there is few in vivo and in vitro *FOXO* deletion experiment focusing on the consequential effect on osteoblast‐specific OCN. In Rached's study, the expression of OCN in *FOXO1ob^–/–^* mice was revealed to increase by 50% in osteoblasts,^30^ suggesting an inhibitory effect of FOXO1 on OCN expression in vivo. In vitro, Yang's experimental results were consistent with those of Rached that the overexpression of *FOXO1* led to a consequential decrease in OCN expression in osteoblast precursors.^32^ However, a contradictory result, knockdown of FOXO1 reducing OCN expression, was reported in another article. And the opposite phenomena may result from different cell culture contexts.^32^, ^53^ Generally, FOXO1 may inhibit OCN expression.

FOXO1 was shown directly to bind to the promoter and the first intron of*Bglap2*, which was the OCN gene, and inhibit its transcriptional activity in COS‐7 cell lines.^30^ However, similar binding in osteoblast cell lines has not been reported yet. Instead, FOXO1 was widely known to take part in Runx2‐dependent and ATF4‐dependent pathways in *Bglap2* transcription regulation in osteoblast precursors. As is known, Runx2 is an osteoblastic‐specific transcription factor known to increase OCN expression by specifically interacted with a chromatin fragment of the proximal *Bglap2* promoter that contains the Runx2‐binding site^32^ (Figure 2). FOXO1 suppressed the interaction between the Runx2 protein and the *Bglap2* promoter, which was proven in loss‐ and gain‐of‐function experiments. When *FOXO1* was overexpressed or knock down in osteoblasts, a decrease and an increase in the interaction between Runx2 and *Bglap2*, accompanied by a decrease and an increase in the transcriptional activity of *Bglap2*, were respectively observed.^32^ As to transcription factor ATF4, it could also interact with *Bglap2* promoter like Runx2, but consequent ATF4‐dependent *Bglap2* transcription activity turned out to be not suppressed by FOXO1.^32^ Moreover, FOXO1 was shown to interact with ATF4 and promote ATF4 activity,^20^ which may suggest an explanation for FOXO1 upregulation OCN in Siqueira's in vitro experiments.^32^, ^53^

Taken together, these results suggest that FOXO1 may balance OCN expression in a two‐tiered mechanism involving interrupting the Runx2‐enhanced transcriptional activity of *Bglap2* and promoting ATF4‐enhanced transcriptional activity of *Bglap2*. Moreover, the former one plays a more dominant role in osteogenesis,^32^ while the latter one refers to ATF4 is still a hypothesis and remains to be further tested.

#### 3.4.6 FOXO1 interacts with ATF4 to enhance formation of mineralized matrix by regulating protein synthesis, oxidative stress and OCN gene transcription

The transcription factor ATF4 is an integral component of a negative‐feedback pathway controlling the import of amino acids and the consequential synthesis of glutathione, which is necessary for the formation of mineralized matrix. Via immunehistochemical analysis, FOXO1 and ATF4 were confirmed to be physically associated and co‐localized in the cytoplasm and nucleus.^20^ When stimuli are absent, the two transcription factors are predominantly located in the cytoplasm. Stress signals stimulate their translocation to the nucleus, where they actively initiate the transcriptional events that protect cellular functionality. This interaction was revealed to promote both FOXO1 and ATF4 activity (Figure 2), like FOXO1‐targeted gene transcription and ATF4‐mediated protein synthesis (glutathione and collagen).^20^ In turn, FOXO1 ablation interrupts the activity of ATF4 and then compromises glutathione and collagen synthesis.^20^ The former one leads to a subsequent increase in ROS, and the latter one results in a decrease in osteoid surface mineralization.^20^ Moreover, *OCN* is also an ATF4 target gene in osteoblasts,^69^ which may provide another explanation for the significant reduction of upregulated OCN expression during osteogenic induction when *FOXO1* was knocked down by siRNA or shRNA.^53^ Collectively, FOXO1 enhances formation of mineralized matrix by interacting with ATF4 and promoting ATF4‐mediated protein synthesis and oxidative stress control in osteoblasts.

### 3.5 FOXOs mediate apoptosis in osteoblasts

Apoptosis is necessary in bone remodelling and plays a critical role in maintaining skeletal homoeostasis, especially after the completion of osteoblasts differentiation.^70^ In vitro, FOXO1/3 and a pro‐apoptotic molecule, Bad, were expressed in primary osteoblasts and the osteoblastic cell line MC3T3‐E1.^71^ Bcl‐2‐interacting mediator of cell death (Bim) is another important regulator of osteoblast apoptosis. Apoptotic stimuli, such as hypoxia, serum deprivation, oxidative stress, radiation and growth factor, upregulate Bim expression in osteoblasts, leading to their apoptosis.^72^ Serum deprivation induces the nuclear entry of FOXO3, which in turn transcriptionally increases Bim expression. Conversely, FOXO3 ablation attenuates Bim expression.^71^ Akt mediates phosphorylation of FOXO1/3 and prevents its nuclear translocation, which suppresses the transactivation of its target gene Bim in osteoblasts.^71^, ^73^ Thus, nucleus translocation of FOXO3 may promote apoptosis by targeting Bim.

Oxidative stress, stimuli of apoptosis, could result from deletion of FOXO1/3/4 in mice. And oxidative stress‐induced apoptosis of osteoblast and osteocyte may be, at least partly, responsible for accompanied decrease in bone mass.^12^ In turn, overexpression of *FOXO3* in vivo, similar to the antioxidants NAC, prevents oxidative stress‐induced apoptosis, further strengthening the contention that FOXO3 exerts antiapoptosis actions in osteoblasts.^12^

In general, FOXO3 may prevent osteoblasts from oxidative stress‐induced apoptosis, while the nuclear translocation of FOXO3 may play a pro‐apoptosis role in osteoblasts.

### 3.6 MicroRNAs mediate osteogenic differentiation by targeting FOXO members

MicroRNAs (miRNAs), endogenous noncoding, single‐stranded RNAs containing 21 to 23 nucleotides, negatively regulate post‐transcriptional gene expression by directly binding to the 3’‐untranslated regions (3’UTRs) of target messenger RNAs (mRNAs).^74^, ^75^, ^76^ Alterations in miRNA expression are closely related to various bone diseases,^77^, ^78^ and miRNAs may be therapeutic targets for the treatment of bone diseases. A group of FOXO‐sensitive miRNAs, including miR‐182, miR‐183 and miR‐705, have also been reported to be crucial regulators of osteogenic differentiation by targeting FOXOs.^79^

Based on the relatively high expression of miR‐182 among miRNAs at the early stage of osteogenic differentiation,^80^, ^81^ miR‐182 was regarded to play an important role in bone formation. However, no consensus target sequence for miR‐182 in essential transcriptional factors for osteogenic differentiation, such as Runx2 and osterix, has been found. This phenomenon was not well‐explained until the FOXO1 3’UTR was confirmed to be a direct target of miR‐182.^13^ This binding leads to inhibition of FOXO1 expression^78^, ^82^ and a consequential decrease in bone formation. Conversely, via inhibiting the PI3K/Akt pathways, miR‐182 assists in the activation of FOXOs,^13^ resulting in an improvement in osteogenesis. Thus, miR‐182 directly inhibits and indirectly promotes osteogenic differentiation. Since the expression of FOXO1 was increased during osteogenic differentiation,^40^ we propose that the high expression and miR‐182‐assisted activation of FOXO1 may override the 3′UTR‐binding‐dependent inhibition of miR‐182 during differentiation. This hypothesis was further supported by Kim's study reporting that overexpression of *FOXO1* overcame the 3’UTR‐binding‐dependent differentiation‐inhibiting effects of miR‐182.^13^

By acting as a sponge of miRNA‐182, osteogenesis impairment‐related long noncoding RNAs (IncRNAs) of human periodontal ligament stem cells (hPDLCs) from periodontitis patients (IncRNA‐POIR) competed with mRNA for binding to miR‐182 and thus antagonized the 3’UTR‐binding‐dependent inhibitory effects of miRNA‐182 on FOXO1.^83^ In this way, IncRNA‐POIR increases the osteogenic differentiation of hPDLCs from periodontitis patients.

By targeting endogenous human FOXO1, miR‐183 negatively regulates cellular proliferation and positively regulates cellular invasion.^47^ It seems that only the human FOXO1 3’UTR contains a functional miR‐183 site, and mouse FOXO1 cannot be regulated by miR‐183.^47^ Human FOXO1 was found to contain two sites predicted to confer miR‐183‐mediated post‐transcriptional regulation: one specific to humans and the other conserved.^47^

In addition, miR‐705 expression was observed to be increased in both osteoporosis BMMSCs and ovariectomy bone tissues by microarray analysis. Via loss‐ and gain‐of function assays, it was revealed that miR‐705 deficiency increased FOXO1 protein accumulation, while overexpression of miR‐705 significantly decreased FOXO1 protein levels. Thus, miR‐705 was predicted to target the FOXO1 mRNA 3’UTR and acts as a novel regulator of FOXO1 via post‐transcriptional regulation in BMMSCs.^84^

## 4 CONTEXT‐DEPENDENT POST‐TRANSLATIONAL MODIFICATIONS OF FOXOS: THE ACTIVATION OR SILENCING OF FOXOS

FOXO transcription factors precisely integrate cellular signals emanating from insulin, growth factors, cytokines and oxidative stress via a combination of post‐translational modifications. Sites of these post‐translational modifications are often located within the conserved regions of FOXO molecules, including the forkhead DNA‐binding domain, the region containing the nuclear localization signal, the N‐terminal region surrounding the first Akt phosphorylation site and the part of the C‐terminal transactivation domain.^15^ These post‐translational modifications alter FOXO intracellular localization, turnover, transactivation and transcriptional specificity and constitute a FOXO “code,” making FOXO1, FOXO3 and FOXO4 highly versatile in gene regulation.^15^ However, the precise mechanisms by which post‐translational modifications regulate FOXO functions are mostly elusive, but in many cases, they seem to affect the DNA‐binding potential of FOXO proteins, the functions of their nuclear localization signal (NLS) and nuclear export signal (NES), or the interactions of FOXO with other proteins. Combinations of upstream imputs alter the post‐translational modification state of FOXO, and these changes control abundance, subcellular localization and DNA‐binding capacity.^85^

### 4.1 FOXO activity is inhibited by insulin‐like growth factor 1 (IGF1)/insulin via the PI3K/Akt pathway

A broad consensus has been reached on the PI3K/Akt signalling pathway being a basic and classic level of FOXO regulation. The Akt‐mediated phosphorylation of FOXO molecules creates two binding sites for the 14‐3‐3 proteins, and the binding complex is then translocated to the cytoplasm, where the bound 14‐3‐3 protein prevents the re‐entry of FOXOs into the nucleus, likely via interfering with the function of their NLS. FOXO1, FOXO3 and FOXO4 are phosphorylated at several highly conserved phosphorylation sites (Thr‐24, Ser‐256 and Ser‐319 for FOXO1^32^; Thr‐32, Ser‐253 and Ser‐315 for FOXO3^86^; and Thr‐28, Ser‐193 and Ser‐258 for FOXO4^87^) via the PI3K/Akt‐dependent pathway. The phosphorylation of these sites adds a negative charge to the positively charged basic region, thereby disrupting the function of the NLS, resulting in its nuclear exclusion and inhibition of target gene expression .^20^, ^88^, ^89^, ^90^ When Akt is inactive and FOXO1/3/4 are dephosphorylated, the NLS may function effectively and thus promote the translocation of FOXO1/3/4 to the nucleus, thereby promoting target gene expression.

**Figure 3:**
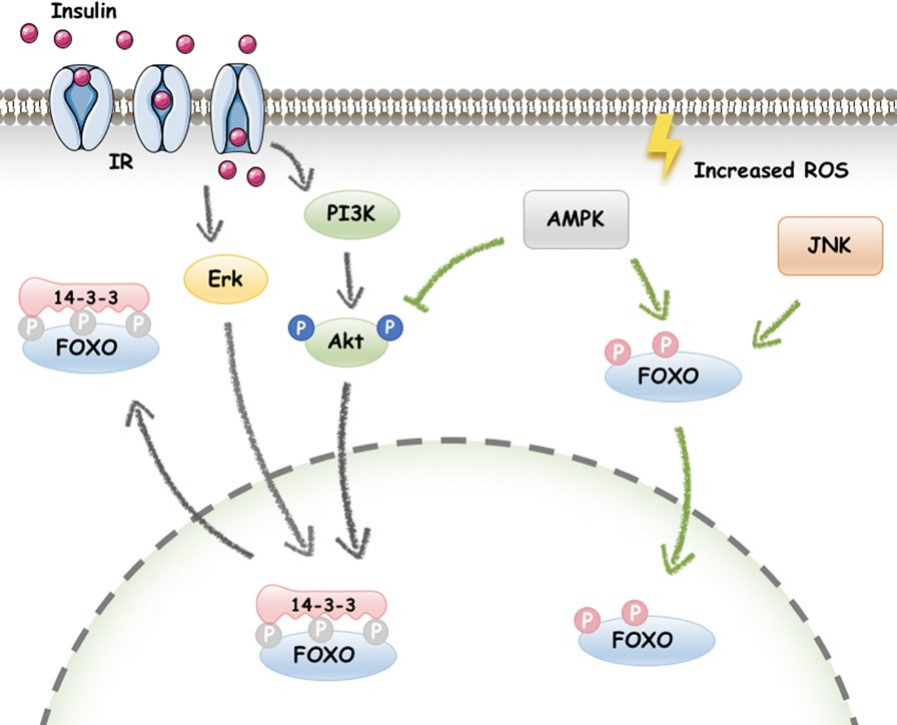
FOXOs is activated or silenced by signalling pathways. A, FOXOs are silenced by IGF1/insulin signalling. IGF1/insulin phosphorylates Akt, and activated Akt triggers the phosphorylation of FOXOs (Thr‐24, Ser‐256 and Ser‐319 for FOXO1; Thr‐32, Ser‐253 and Ser‐315 for FOXO3; and Ser‐193 and Ser‐258 for FOXO4). The AKT‐mediated phosphorylation of FOXO induces binding of 14‐3‐3 proteins, and the resulting complex is then translocated into the cytosol, where the bound 14‐3‐3 protein prevents re‐entry of FOXOs into the nucleus, likely by interfering with the functions of their NLS. B, AMPK signalling reverses PI3K/Akt‐inhibited FOXO activity under stress. AMPK dephosphorylates Akt and directly phosphorylates FOXO3 at its C‐terminal domain, inducing the nuclear translocation of FOXO. C, Erk, like Akt, is also preferentially activated in response to growth factors. And phosphorylation by Erk inhibits FOXO activity by promoting its nuclear export to cytoplasm or proteasome‐mediated degradation. D, JNK, a MAPK family member activated by stress stimuli, is responsible for FOXO activation under stress conditions

It is known that diabetes is a secondary cause of osteoporosis, and IGF1/insulin signalling is reported to regulate postnatal bone remodelling via the PI3K/Akt pathway, as described above.^88^, ^91^ IGF1, which is a growth factor, could phosphorylate Akt and thereby inactivates FOXO transcription factors. The IGF1 receptor and insulin receptor are both expressed in osteoblasts, and specific deletion of insulin receptor severely impairs osteogenic differentiation.^88^ This impairment was explained by an IGF1/insulin/Akt/FOXO pathway proposed by Stitt.^92^ In this theory, IGF1/insulin phosphorylated and exported FOXO1 from the nucleus to the cytoplasm via the PI3K/Akt pathway (Figure 3). Then, IGF1/insulin‐induced phosphorylation of FOXO1 was found to enhance its ubiquitination, helping to target FOXO1 for degradation.^93^ Further, the IGF1‐induced translocation of FOXO1 to the cytoplasm was observed to be abolished by PI3K/Akt inhibition, which could result from the AMPK pathway.^94^

In turn, overexpression of constitutively active FOXO1 significantly upregulated the abundance of IGF1 receptor, insulin receptor and IGF1‐induced Akt phosphorylation,^95^ suggesting that FOXO1 may provide feedback by promoting insulin and IGF1 receptor signalling via PI3K/Akt pathway.

### 4.2 AMPK reverses PI3K/Akt‐inhibited FOXO activity under oxidative stress

AMP‐activated protein kinase (AMPK) pathway, which can be activated by conditions such as oxidative stress and glucose starvation, is an indicator of energy depletion that induces oxidative metabolism in addition to regulating mitochondrial biogenesis, autophagy, cell growth and proliferation.^96^ AMPK was found to be capable of reducing cellular stress and increasing cell survival via direct phosphorylating and activating of FOXO1^97^ and FOXO3.^98^, ^99^ By directly phosphorylating human FOXO1/3 in mammalian cells, AMPK promotes FOXO1 nucleus translocation, establishing the molecular basis for the increase of FOXO1 stability (by escaping proteasomal degradation) and FOXO1/3‐dependent transcriptional activity (such as manganese superoxide dismutase and catalase).^97^, ^98^ Although the AMPK‐FOXO axis has only been tested in several cell lines rather than osteoblast cell line,^98^ increasing evidences suggest that AMPK activation stimulates the differentiation and mineralization of osteoblastic MC3T3‐E1 cells,^94^, ^100^, ^101^ indicating a potential role of AMPK‐FOXO axis in osteoblasts. Moreover, AMPK activation was proven to induce de‐phosphorylation of Akt and consequently reversed the PI3K/Akt‐inhibited FOXO activity (Figure 3) and osteogenesis.^94^, ^102^ Thus, in response to oxidative stress, AMPK may enhance osteogenesis by directly binding to FOXO1/3 factors or indirectly de‐phosphorylating of Akt. And the former one needs to be further tested in osteoblasts.

### 4.3 Cellular stresses‐activated Mitogen‐activated protein kinase (MAPK) cooperates/antagonizes with Akt on the nuclear translocation of FOXO

Mitogen‐activated protein kinase (MAPK) family members, including extracellular signal‐regulated protein kinase (Erk), c‐jun NH2‐terminal kinase (JNK) and p38 MAPK (p38), mediate a wide variety of cellular processes in various cells in response to extracellular stimuli.

Typically, FOXO factors become phosphorylated and localized in the cytoplasm in response to survival and growth factors. FOXO is negatively regulated by a number of survival signalling pathways, such as Erk and Akt.^103^ Like Akt, Erk is also preferentially activated in response to growth factors.^104^ Phosphorylation by Akt and Erk inhibits FOXO activity by promoting its nuclear export or proteasome‐mediated degradation.^105^ And the phosphorylation promotes FOXO1/3 nuclear‐to‐cytosolic translocation.

JNK is responsible for FOXO activation under stress conditions.^106^ Early evidences have shown that growth factor‐activated Akt and stress‐activated JNK have opposing effects on FOXO; Akt prevents FOXO1/3/4 nuclear localization and inhibits its activity, whereas JNK increases FOXO1/3/4 activity by promoting its import into the nucleus of stem cells.^107^, ^108^ Furthermore, Akt inhibition was proven to lead to increased JNK phosphorylation in islet cells, and this could be reversed by the specific JNK inhibitor SP600125.^109^ JNK could directly phosphorylate FOXO4 at T447 and T451, inducing FOXO4 activation and retaining FOXO4 in the nucleus in NIH3T3 cells.^107^ Additionally, JNK was proven to phosphorylate 14‐3‐3 at S184^110^ and then release FOXO to enter the nucleus in colon cancer cells.^111^ Thus, nuclear translocation of FOXO can be regulated via the JNK‐Erk/Akt pathway in mammalian cells.

Generally, Erk and JNK are essential in regulation of FOXO activities in various mammalian cells. As to stem cells, JNK phosphorylates and activates FOXOs in response to oxidative stress, while Erk phosphorylates and nuclear‐excludes FOXOs for their attenuated transcriptional activities in response to growth factor. And few bone research mentions about p38/FOXO axis.

### 4.4 Inflammation activates FOXO1/3, while FOXO activation antagonizes inflammation‐induced bone resorption

Pathogenic signals from oxidative stress, inflammatory mediators and dysfunctional cell signalling trigger inflammation. Studies in patients with rheumatoid arthritis and osteoarthritis following synovial biopsy have demonstrated the phosphorylation of FOXO1/4 in macrophages and FOXO3 in lymphocytes, indicating that loss of functional FOXOs may lead to inflammatory cell activation in these disorders.^112^

Inflammation was proven to increase FOXO3 expression in vascular smooth muscle cells^113^ and periodontal ligament stem cells, suggesting an activation role of inflammation to FOXO3. Fracture healing was delayed by diabetes‐induced TNFα in MSCs, accompanied by an upregulation of FOXO1 nuclear translocation.^39^ This phenomenon indicates TNFα may active FOXO1. This hypothesis was further confirmed in Almeida's study that TNFα‐mediated activation of FOXOs in osteoblasts was revealed via a ROS/JNK signalling cascade.^68^ And activated FOXO1/3 turn out to play an antiinflammatory role to reverse inflammation‐induced bone resorption.

According to the limited studies reporting about the affection of inflammation on FOXOs in bone, we know that inflammation activates FOXO1/3 and FOXO1/3 may serve as sensors of inflammation‐induced cellular stress in turn.

Thus, AMPK and JNK phosphorylate and activate FOXOs, while Akt and Erk phosphorylate FOXOs and exclude them from the nucleus to attenuate FOXOs transcriptional activities.^114^ In addition to PI3K/AKT‐, AMPK‐ and MAPK‐mediated phosphorylation, the function of FOXO proteins is also controlled by other types of post‐translational modifications, including acetylation by calcium response element‐binding (CREB)‐binding protein (CBP),^114^ methylation by arginine methyltransferase PRMT1^115^ and O‐linked glycosylation by glucosamine.^41^, ^116^, ^117^

## 5 CONCLUSION

**Figure 4:**
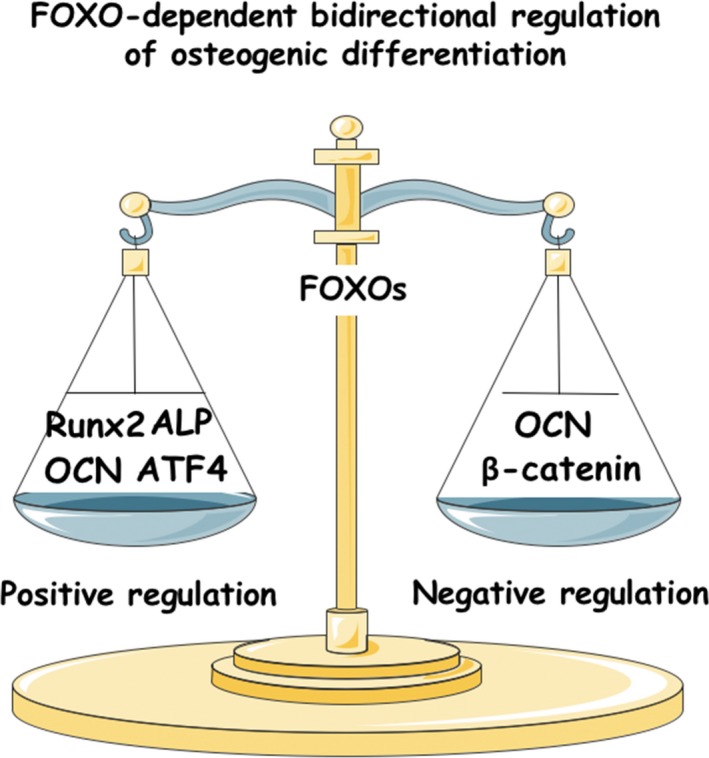
FOXOs balance the positive (via *Runx‐2*,* ALP*, OCN, ATF4) and negative (via β‐catenin, OCN) regulation of osteogenic differentiation in MSCs

By initiating differentiation, modulating lineage commitment and affecting mineralization, FOXO1/3/4 appear to control the generation from MSCs to early progenitors, to osteoblast precursors, to mature osteoblasts and then to osteocytes. FOXO factors may fulfil a critical function in balancing positive (via *Runx‐2*,* ALP*, OCN and ATF4) and negative (via β‐catenin and OCN) regulation of this progression (Figure 4). As individual of osteogenic‐regulatory factors takes in charge in different differentiation‐degree osteogenic lineages (like Runx2 in early progenitors, β‐catenin in osteoblast precursors, and OCN, ALP and ATF4 in mature osteoblasts), FOXOs may also change their roles by interacting with these factors throughout differentiation. Thus, FOXOs may promote osteogenesis in early progenitors and mature osteoblasts, while inhibit that in committed osteoblast precursors. Different models of signalling inputs transduced from contexts (oxidative stress, growth factor and inflammation), including PI3K/Akt signalling, AMPK signalling, MAPK signalling and miRNAs, may also shift the balance. The development of pharmacological agents targeting FOXOs may shed light upon treatment of bone formation defect diseases. Before that, several questions should be paid attention to first. What is the complete and precise mechanism of FOXOs performing role transitions between promoting and inhibiting osteogenesis along differentiation? And how do additional contexts affect this process? The main gap in our understanding today lies in the cell‐specific, molecular mechanisms of the context regulation of FOXO‐dependent osteogenic differentiation both at transcription level and post‐translational level. As to a single context like inflammatory microenvironment, its affection on FOXOs was mainly studied in macrophages, T cells and tumour cells, and further exploration in osteoblasts is required.

## CONFLICT OF INTERESTS

The authors declare no conflict of interest.
